# Imagining Kant’s theory of scientific knowledge: philosophy and education in microbiology

**DOI:** 10.1007/s10123-022-00315-z

**Published:** 2022-12-23

**Authors:** Fernando Baquero

**Affiliations:** Department of Microbiology, Ramón y Cajal University Hospital, and Microbial Biology and Evolution Area of the Ramón y Cajal Institute for Health Research (IRYCIS), Madrid, Spain

**Keywords:** Kant, Critique pure reason, Science Education, Evolutionary epistemology, Microbiology as science

## Abstract

In the field of observational and experimental natural sciences (as is the case for microbiology), recent decades have been overinfluenced by overwhelming technological advances, and the space of abstraction has been frequently disdained. However, the predictable future of biological sciences should necessarily recover the synthetic dimension of “natural philosophy.” We should understand the nature of Microbiology as Science, and we should educate microbiology scientists in the process of thinking. The critical process of thinking “knowing what we can know” is entirely based on Kant’s Critique of Pure Reason. However, this book is extremely difficult to read (even for Kant himself) and almost inaccessible to modern experimental natural scientists. Professional philosophers might have been able to explain Kant to scientists; unfortunately, however, they do not get involved this type of education for science. The intention of this review is to introduce natural scientists, particularly microbiologists and evolutionary biologists, to the main rigorous processes (aesthetics, analytics, dialectics) that Kant identified to gain access to knowledge, always a partial knowledge, given that the correspondence between truth and reality is necessarily incomplete. This goal is attempted by producing a number of “images” (figures) to help the non-expert reader grasp the essential of Kant’s message and by making final observations paralleling the theory of scientific knowledge with biological evolutionary processes and the role of evolutionary epistemology in science education. Finally, the influence of Kant’s postulates in key-fields of microbiology, from taxonomy to systems biology is discussed.

## Introduction

Maureen A. O’Malley, from Sidney University, published in 2014 a seminal book entitled “Philosophy of Microbiology.” In the first words of her introduction, she states that “there are many good reasons to think that in fact microbes form the bases of all biological things and thus have major contributions to make to philosophy of biology.” She recognizes Aristotle (384–322 BCE) and Kant (1724–1804) as the philosophers most invoked in the philosophical tradition of reflecting on the nature of living things. Note that Immanuel Kant was born almost exactly one century after Anton van Leeuwenhoek (1632–1723), and thus belongs to the “microbiological era.” We are very close to commemorating the 3^rd^ centenary of the birth of Immanuel Kant on the 22^nd^ of April 1724 in Königsberg (now Kaliningrad, Russia) where he died in 1804. Charles Darwin was born just 5 years later in 1809. In 1904, the German evolutionary biologist Ernst Mayr was born in Kempten, Germany. Mayr died in 2005, 200 years after Kant. Only these two names are sufficient to bridge our days with the Kant’s days.

Kant’s powerful shadow extends even over until modern’s evolutionary biology. In one of his latest published books, “This is Biology” (Mayr 1997), Ernst Mayr quotes Charles Darwin 22 times and Immanuel Kant 13 times, more than other highly recognized biologists such as Linnaeus, Haeckel, and Lamarck. Indeed, Kant was one of the last representatives of classical science, where philosophy and natural sciences were still in permeable compartments of scientific knowledge. An entire generation of Kant’s immediate German followers can be properly considered to be philosopher-scientists, such as the botanist Matthias Jakob Schleiden (1804–1881), who developed “modern science” in researching vegetable tissues, and Jakob Friedrich Fries (1773–1843), one of the pioneers of modern thinking in language and science. It is such permeability between compartments of knowledge that is being lost in modern times. Ernst Mayr was already disappointed in his youth when he attended philosophy courses at the Berlin University in the early 1920s, realizing that there were “no bridges between the matter of study of biological sciences and that of philosophy,” coining the idea that evolutionary biology, developed from empiric (scientific) knowledge, is based on concepts rather than laws.

Such statement is probably rooted in Kant’s philosophy of knowledge. Kant’s Theory of Empiric Knowledge is essentially expressed in the Critique of Pure Reason (CPR), which he began to conceive in 1772, published in 1781 (“A”) and modified in 1787 (“B”) (Kant 1787). The Critique is a difficult-to-read book that clearly expressed the evolution of Kant’s mind during the writing process and was therefore not devoid of a certain number of obscurities, inconsistencies, contradictions, and reiterations but remains extremely engaging, even passionate, and frequently less boring (at least for a scientist) than many other books that commented on Kant’s works in academic detail.

On what grounds would a review of Kant’s Theory of Empiric Knowledge be of interest for modern biologists? First, this theory constitutes one of the highest peaks reached by the human mind (“because it is there,” paraphrasing George Mallory reason to climb the Everest), and science is based on thinking. As natural scientists, we should urgently emphasize the importance of the creative power of individual thinking when considering natural empirical facts. Second, the theory constitutes a paradigmatic example of individual introspective research, examining our ability to understand nature. Lastly, Kant’s Theory of Empiric Knowledge can be properly considered (according to Heidegger) as a theory of scientific knowledge (Huttunen and Kakkori 2020). In this theory, knowledge enables us to understand or even conceive something as different from another and what, based on that conception, is needed to establish an assertion, proposition, or judgement. This is exactly the purpose of science: the origin of the word “science” is probably originally related with “*scindere*” (to cut something to understand the internal structure).

Living beings constitute ordered, combinatorial, architectural alternatives to the chaotic multiplicity of elements of nature. Similarly, human reason is an ordered, combinatorial, architectural “internal” alternative to the immense wealth of inputs that we receive from our environment. The architecture of nature should have a “similar style of architecture” as that of our mind (the Aquinean “*ars imitatur naturam in sua operatione*” [art imitates nature by reproducing it]). Interestingly, Kant clearly states that “human reason is by nature architectonic” (CPR, A473/B501). It is only through this “common style” that the ordered part of nature might be understood, at least within the boundaries imposed by our psychological and biological limits. Understanding nature indeed requires to understand what we can understand. Nature can be conceived as what we understand about Nature: “The order and regularity in appearances, *which we call nature*, are, then, something that we ourselves supply, nor we encounter them if we, *or the nature of our mind*, had not originally supplied them” (CPR, A125). Certainly these basic nature-mind unitarian concepts are implicitly present throughout Kant’s Theory of Knowledge. The basic aim of understanding nature is to produce science, that is, not only to discover or experience things but in particular “experiencing things that one can go on to describe.” A faithful description is the result of a chain of quality controls in the process of experiencing and understanding, leading to a final synthetic knowledge, a knowledge able to be communicated, rooted in nature but different from natural empirical objects (that is, “a priori”). The entire CPR is devoted to investigating the conditions that are needed to reach such a knowledge, as expressed in its central question, “How is synthetic a priori knowledge possible?” What does the question “how is science possible?” imply? This review illustrates the main structures of the architectural design of the theory of knowledge. “By the term architectonic I mean the art of constructing a system. Without systematic unity, our knowledge cannot become science; it will be an aggregate, and not a system,” says Kant (CPR A831), as an unequivocal prelude to modern systems biology, being amazingly compatible with current views in the field of neurosciences. How can objective things be thinkable? How to convert something external, physical, or objective into a concept or idea? How to apply to it concepts arising from other external things?

## The educational value of thinking about what we can know

A key work in the theory of education is *Education* by Herbert Spencer, a prominent follower of Darwin (Dewey 1904). Spencer answers the question “what knowledge is of most worth?” with a single word: science (Spencer 1924). Spencer’s novel philosophical approaches are likely grounded in the post-Kantian philosophy of Germany, despite Spencer’s opposition to Kant’s apparent supernaturalism. Spencer was the first to use the concept of “survival of the fittest” (1851), which was later adopted by Darwin. Dewey noticed a footnote in Spencer’s book *Social Statics*, probably based on the post-Kantian Friedrich Shelling, stressing the natural tendency towards individuation, conjoined with increased mutual dependence. In *The Classification of the Sciences*, which Spencer realized that this truth has to do with “a trait of all evolving things, inorganic as well as organic” (Dewey 1904). Performing science is an educational tool for understanding the functioning of one’s own mind, given that the roles of understanding are essentially evolutionary roles, individuating the objects and synthesizing their ensembles to reveal their mutual dependence. Kant should serve as a “teacher of biology” (Rabel 1931).

In current technological days, educating the minds of scientists is still the best strategy to advance science, but the principle of Wittgensteinian objectivity should be present; we know and will know what we can know and nothing more (Baquero and Moya 2012). From this viewpoint, we can try to use educational tools to push the limits, that is, to increase our ability to know (Fischer et al. 2014), in line with the lines of modern evolutionary epistemology.

In this article, we present Kant’s Theory of Knowledge in an accessible (visual) and educational manner, emphasizing the parallelism of knowing and evolving. Readers should, however, be aware of the following caveat. The author of this review is not a professional philosopher but an experimental scientist (microbiologist) and professor who has been thoughtfully analyzing and scholarly discussing Kant’s original contributions for at least four decades. Professional philosophers might be able to explain Kant to scientists; unfortunately, they tend not to get involved in this type of education. Consequently, this educational review is necessarily a simplified, schematic and perhaps slightly inaccurate explanation but will hopefully make one of the more important but complex works that the human mind has produced, the Critique of Pure Reason, accessible to scientists.

## The three successive compartments of knowledge

**Fig. 1:**
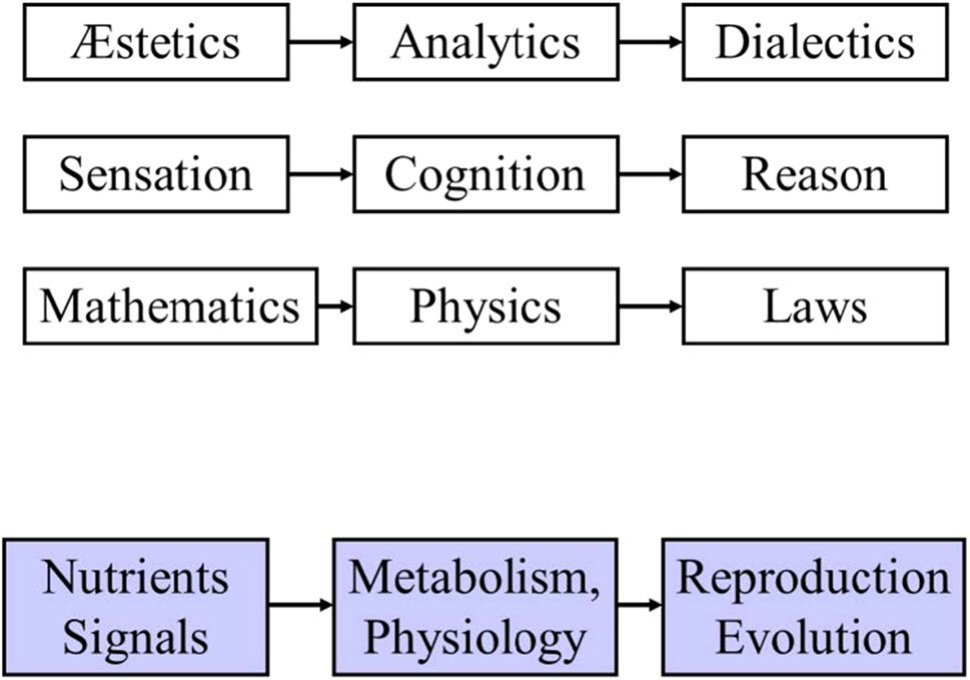
The three consecutive compartments with the conditions for knowledge. In the first row, the Kant’s conditions; below, analogies of these conditions; in violet, biological analogies to the conditions for knowledge, to introduce the relation of knowing and evolving

The CPR section entitled “Reason in General” starts with a clear enunciation of the three main compartments in the process of knowledge: “Everything in our knowledge starts in our **sensibility**; from there, flows into the **understanding**, and finally enters into our **reason** (CPR, A298) (“Alle unsere Erkentnis hebt von den Sinnen an, geht von da zum Verstande und endigt bei der Vernunft, über welche nichts Höheres in uns angetroffen wird…..”). These three compartments are shown in Fig. 1 and refer to the main parts into which the CPR is divided.

First, the conditions by which natural beings are perceived by our sensibility are studied in the **Æstetics**. Second, the conditions by which the impressions (intuitions) that these natural beings produce in our sensibility are converted into concepts, giving rise to their cognition, are studied in **Analytics**. Third, the conditions by which concepts are submitted to relational judgements, making possible the emergence of ideas, are studied in **Dialectics**. All three compartments (the Æstetics, Analytics, and Dialectics) are equally qualified in the CPR as “transcendental,” given that the conditions studied in each of them transcend (encompass) any possible natural (empirical) object and only apply to the a priori conditions of any knowledge. This identification of three successive compartments in the process of knowledge fits well with old-rooted views of scientific common wisdom. The mathematics-physics (natural sciences)-general laws triad expresses the same flow, and biologists will recognize analogies with our familiar cascade (nutrient and signal recognition/uptake-metabolism-reproduction/evolution].

### The first compartment: Æstetics

**Fig. 2:**
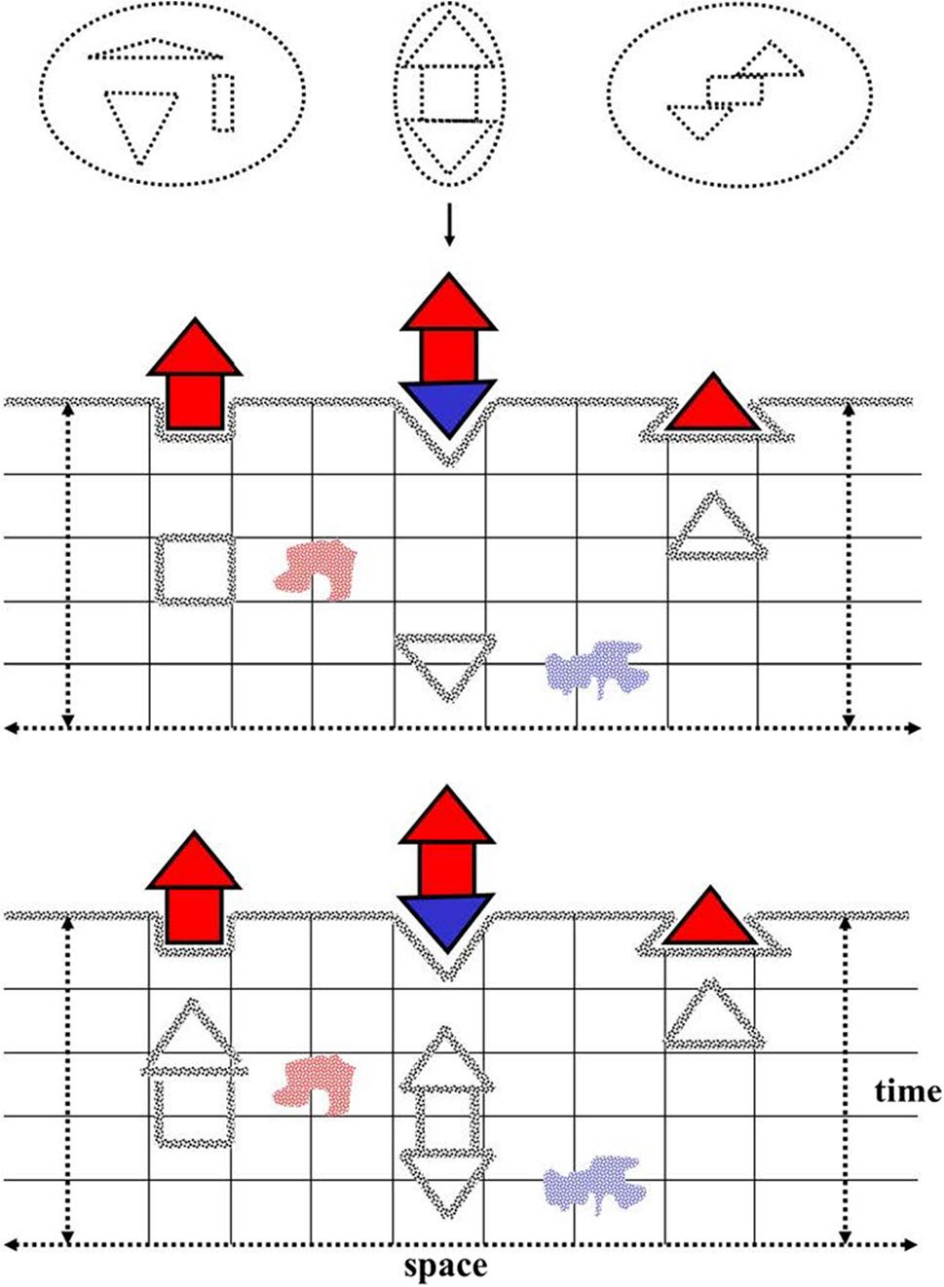
Æstetics, the condition for sensibility. Up in the figure, external “things-in-themselves” than are perceived in our sensibility by the deformation of an “a priori” space–time dimensional field in our mind. Below, two successive instants where these “things-in-themselves” are perceived as eventually composed by parts, with different intensities or dimensions, that is, we have an “intuition” of them

**Æstetics** is the compartment of human sensibility. We prefer not to use the term “senses” (*Sinnen*) here as in Kant’s original text, given that modern technology has significantly extended the power of our natural “senses” but still provide only elements for our sensibility. To sense something implies the existence of a deformable “membrane” differentiating the outside and the inside but able to connect both sides. The inside should be a mind “receptive for impressions” (CPR, A50/B74), a receptive subject (a “me”) able to be influenced by the outside. The significant outside is composed by the type of external “things” that, reaching our neighborhood, can influence our sensibility. Out of us, natural things remain unknowledgeable in their intrinsic ontological nature, centripetally directed to themselves; they are, in Kant’s words, just “things-in-themselves” or “intelligible existences” or “noumena” (CPR, B306). These things can only influence our internal senses and therefore become visible for our knowledge, when wrapped (the term is mine) *within* our sensibility with space and time. We can imagine, as in Fig. 2, that space and time are two *internal* dimensions providing shape (cognoscibility) to the external things-in-themselves; in other words, what we perceive from external things is just the deformation they produce in our internal space–time frame, resulting in the “**intuition**” of them. A particular place of space–time cannot be filled by identical objects, even if they share identical features. We can imagine these impressions or intuitions of our sensibility as different forms of colors and shapes and as particular intensities and dimensions and occurring in successive instants; at this stage, however, they are nothing like complex “objects” or “things.” The Kantian revolutionary view implies that space and time are not empirical properties associated with the appearance of these external things (phenomena) but just “a priori,” intrinsic, structural conditions of our sensibility. It is the intuitions from things (*qua* subjects of the senses) that conform to the nature of our faculty of intuition, making it possible (as we will see later) for us to think about them. The object is not the source of any form, rather, it is formalized by intuition. Kant was well aware of such a revolution, compared it with the Copernican revolution in the Preface to the Second Edition of CPR (B-XV): “We here propose to do just what Copernicus did in attempting to explain the celestial movements.” Indeed, it was rather an opposite but equivalent movement. With Copernicus, the Earth was displaced from the center of the system; with Kant, the “object”—as primary source of all its objective knowledgeable attributes—was displaced from the center, being replaced by the aprioristic frame of pure reason; everything we know about external things is being produced by ourselves. Obviously, as things are formalized by means of our time and space (as “a priori” structural, essential, pure conditions of our sensibility), we cannot conceive anything represented outside of time or space.

## The second compartment: analytics

**Analytics** is the compartment of our cognition (understanding), which is based on the elaboration of concepts (in German, *Begriffe*) using the material about external things provided by intuitions. “Intuitions without concepts are blind” (CPR A50/B74), which could be better expressed as, “we are blind for intuitions without concepts.” Given that intuitions are based on experience (interaction with external things), concepts derived from this experience are called **empirical concepts** (A220/B267). Paraphrasing Hartnack (Hartnack 2001), an intuition might be just a particular bacterial form with a particular color that is detected under the microscope when examining the liquor of a patient with meningitis but that is only recognized as *Streptococcus* if the observer has an empirical concept of *Streptococcus*. However, the building-up of empirical concepts requires the contribution of other type of concepts, not derived from experience, that is, **“*a-priori*” (pure) concepts**, present in the architectural framework of our understanding. These pure concepts serve to establish relationships between intuitions and previously acquired empirical concepts, giving rise to judgements: this stained form corresponds to a *Streptococcus* (an affirmative judgement). The judgement is the result of applying a pure concept, the concept of “reality” or “identity” (obviously needed to formulate the question: has “A” the same reality, the same identity as “B”?), which links intuition with a previously known empirical concept. Note that intuitions are made by “a priori” conditions of our sensibility (space–time), and “*a-priori*” concepts (such as “reality” and “identity”) convert them, by means of a judgement, into “empirical concepts.”

In the CPR, the pure concepts of understanding are denominated “**categories,**” a possibly puzzling term for modern scientists, especially for evolutionary biologists who will interpret “categories” as ranks or levels in a hierarchic classification; as a class, the member of which are all the taxa to which a given rank is assigned (1). This is an evolved definition of the old classic Aristotelian term “category,” which is essentially the one employed by Kant. In this classic definition, categories are the rules that should be applied to make clearer the “type of thing” we are sensing with intuition, for instance, if it is or not like other things we know or if it is single or multiple, occasional or constant. In other words, the categories are, in the Aristotelian sense, predicaments, serving to link a predicate to the objects provided by the intuition, in a sense, to trigger a first judgement about how these objects appear. The notion of link is critical here. Categories provide “linking power,” in Kant’s words, providing “connections” in a process of “**pure synthesis**” (that is, between a priori, pure elements), i.e., “joining different representations to each other, and comprehending its multiplicity in one act of knowledge” (CPR A77-A80). Thanks to categories, the intuitions are submitted to knowledge: for the first time, they can be thought. To a certain extent, this view parallels what is familiar to us in biochemistry, that categories are functional activities, as “enzymes,” ensuring the binding of different molecules. Applying categories to intuitions results in “**judgements.”**

Kant differentiated 12 “categories” within the Analytics compartment (to a certain extent, just a “round magic number”) that submit the intuitions to qualitative, quantitative, relational, and modal analysis, as they successively appear in the time–space frame of the Æstetics compartment.

**Fig. 3:**
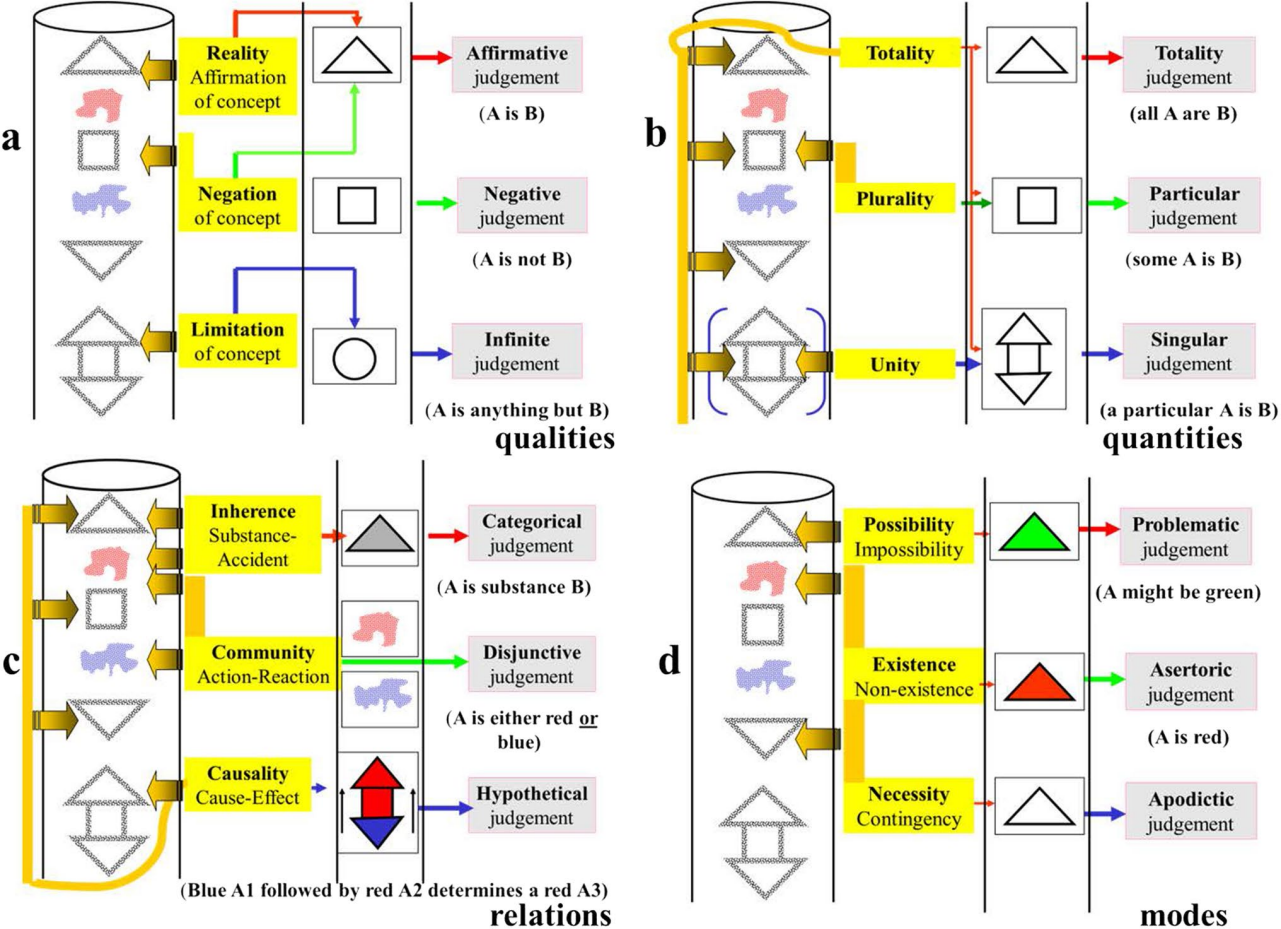
The 12 categories. The “things” or “objects” sequentially captured by our sensibility (vertical tube) are analyzed in their qualities, quantities, relations, and modes by the 12 categories of pure understanding (in yellow) matching them with empirical concepts, giving rise to judgements (in gray). Dark yellow arrows correspond to the “schemas” providing an abstract “image” of what was perceived by sensibility (see Fig. 4)

Figure 3a illustrates the following categories analyzing the **qualities** of intuitions: categories of *Reality* (examining whether we can link an *affirmative* predicate to the intuition; in plain words, if we could think of something affirmative about it), *negative* (if we could think of something denying a particular attribution), and *limitative* (if we could eliminate an attribution among many others). The resulting judgements are respectively *affirmative* judgements (A *is* B), *negative* judgements (A *is not* B), and *infinite* judgements (A *is everything but* B). Figure 3b illustrates the categories analyzing the **quantities** of intuitions. These are the categories of *totality* (could be something postulated –predicated– for all intuitions of this type), *plurality* (could be something predicated for a number –more than one– of these intuitions), and *unity* (could be something predicated just to a single member of the perceived intuitions). The resulting judgements are respectively *totality* judgements (*all* A *are* B), *plurality* judgements (*some of* A *are* B), and *unity* judgements (a *particular* A *is* B). Figure 3c illustrates the function and effect of categories analyzing the **relationships** that can be predicated to the intuitions. These are the categories of *inherence-subsistence* (the intuition corresponds to either a *substance* or an *accident*; substances and accidents are respectively permanent or changing traits and could be conceived as a kind of relationship within the intuition), *community* (a predicate could be applied such that another one is specifically excluded, and vice versa, so that if one acts, the other reacts in a type of antagonistic reactive community), and *causality-dependence* (could be predicated if the intuition is a cause or a consequence of something). The resulting judgements are respectively *categorical* judgements (A is of the *substance* –or accident– *of* B), *disjunctive* (A is *either* A or B), and *hypothetical* judgements (can we attribute a cause-effect A → B determination to a given temporal succession of intuitions A and B?). Lastly, Fig. 3d illustrates the functions of the categories analyzing the **modes** that can be predicated to the intuitions. These are the categories of *possibility* (could we attribute a predicate with a *probability of* being fit –existent– to the intuited?), *existence* (one-step further, could we affirm that it exists?), and *necessity* (could we predicate something that not only applies but should apply necessarily?). The resulting judgements are respectively *problematic* (the affirmation or negation is accepted as merely possible), *assertoric* (we regard the proposition as real or true), and *apodictic* (we look on it as necessary).

We have introduced in Fig. 3, in a parallel manner to the flow of intuition, another flow of “predicates” that are linked to intuitions by categories and provide material to judgements. These predicates should be “**empirical concepts**” that have been collected necessarily as a result of previous experiences. For a modern biologist, there is the temptation to assume that the result of the knowledge process should be the creation of novel empirical concepts, which will enter into the flow to bind new intuitions through the categories and thereby endlessly provide better possibilities for understanding. As stated by Justus Hartnack, “The empiric concept can be considered as a rule to know, to recognize, or imagine, the type of things or objects that the concept represent” (9). That view is poorly expressed both in the CPR and in most comments about the CPR, probably because the main focus for Kant was “pure” reason. Hartnack states, “Obviously there are a countless number of empirical concepts to speak about what is provided to us by intuition. Nevertheless, what matters here are not the empirical concepts, but rather those concepts that are a priori*.*” In any case, “existence” is not considered a predicate.

As we have seen before, there are 12 categories and correspondingly 12 judgements. Kant stated that this series of operations covered all possibilities by which an object arriving from the field of experience (empirical) could be understood by pure reason. We can now see clearly why we emphasize include the term “scientific” in the title of this review. Indeed, Kant’s Theory of Knowledge applies more to the scientific method of thinking (judgements as to whether A is precisely B or not) than to ordinary thinking (what is A?).

## The link between the Æstetics and analytics compartments: the schemas

**Fig. 4:**
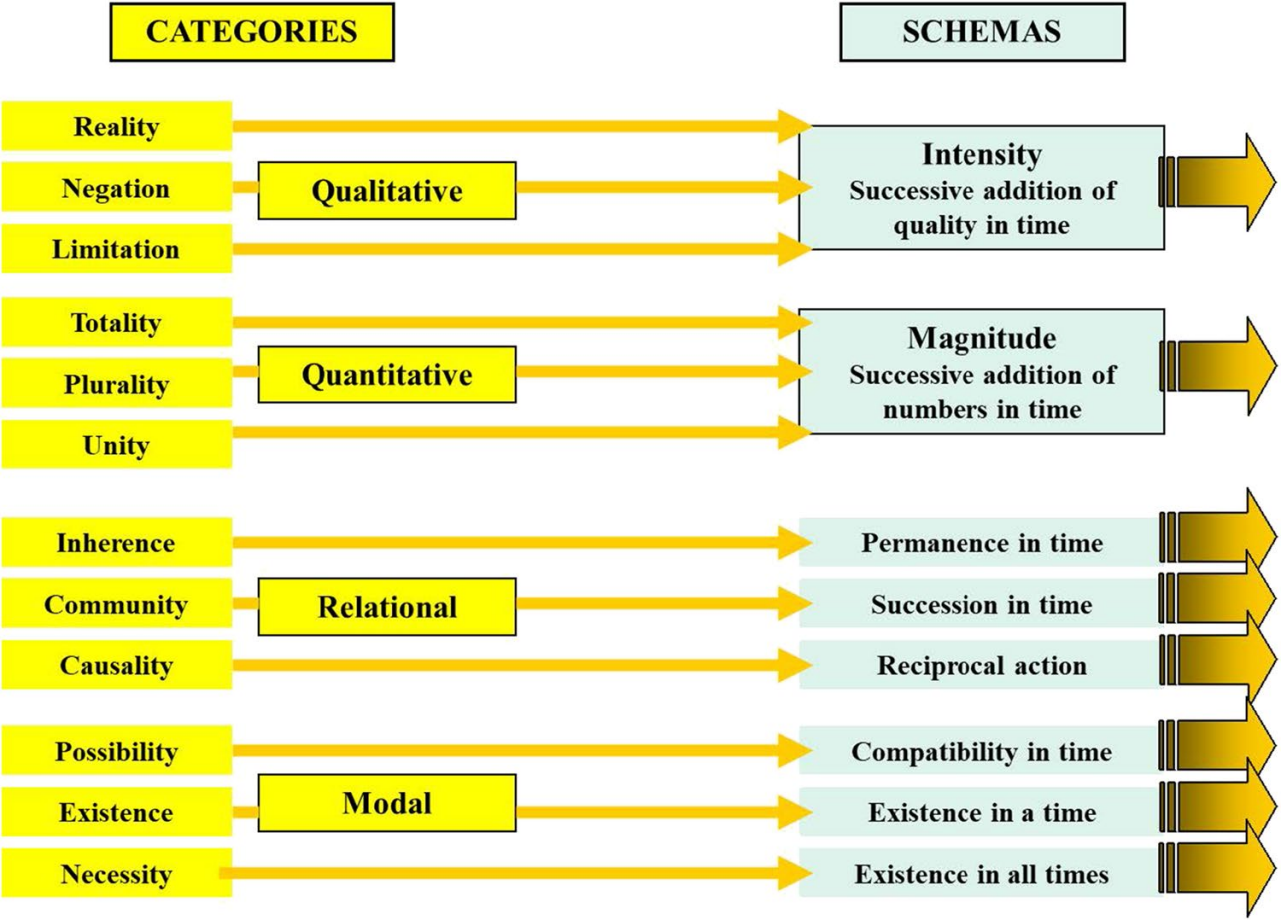
The schemas. The schemas provide an abstract image of the nature of intuitions detected in our sensibility. The schemas (in blue) serve to link (dark yellow arrows) the “categories of understanding” (in yellow) with the intuitions of our sensibility, exclusively based on their “time component” (see Fig. 5). Each of the relational categories has its own schema

How can an intuition, a pure imprint in our sensibility, wrapped in just space and time, be “considered” by the categories, which are “a priori*”* pure concepts, but ultimately concepts, thus necessarily outside of space and time? This was a critical problem that Kant solved by introducing the notion of “schemas.” Categories, when entering into timeframe-wrapped intuitions, conform themselves into “schemas” (“schematism of pure concepts of understanding”). The schemas are an abstract image of the intuitions, exclusively based on their “time component.” We could say that the time component of intuitions determines something like an imprint in the pure tissue of categories. In an extremely abstract Kantian view, time (our “internal time,” but only time) is sufficient to describe (to imagine, to illustrate) any empirical object. It is for this reason that “time” was considered an absolute “a priori” condition of knowledge. As illustrated in Fig. 4, different types of categories adopt the form of different types of schemas.

**Fig. 5:**
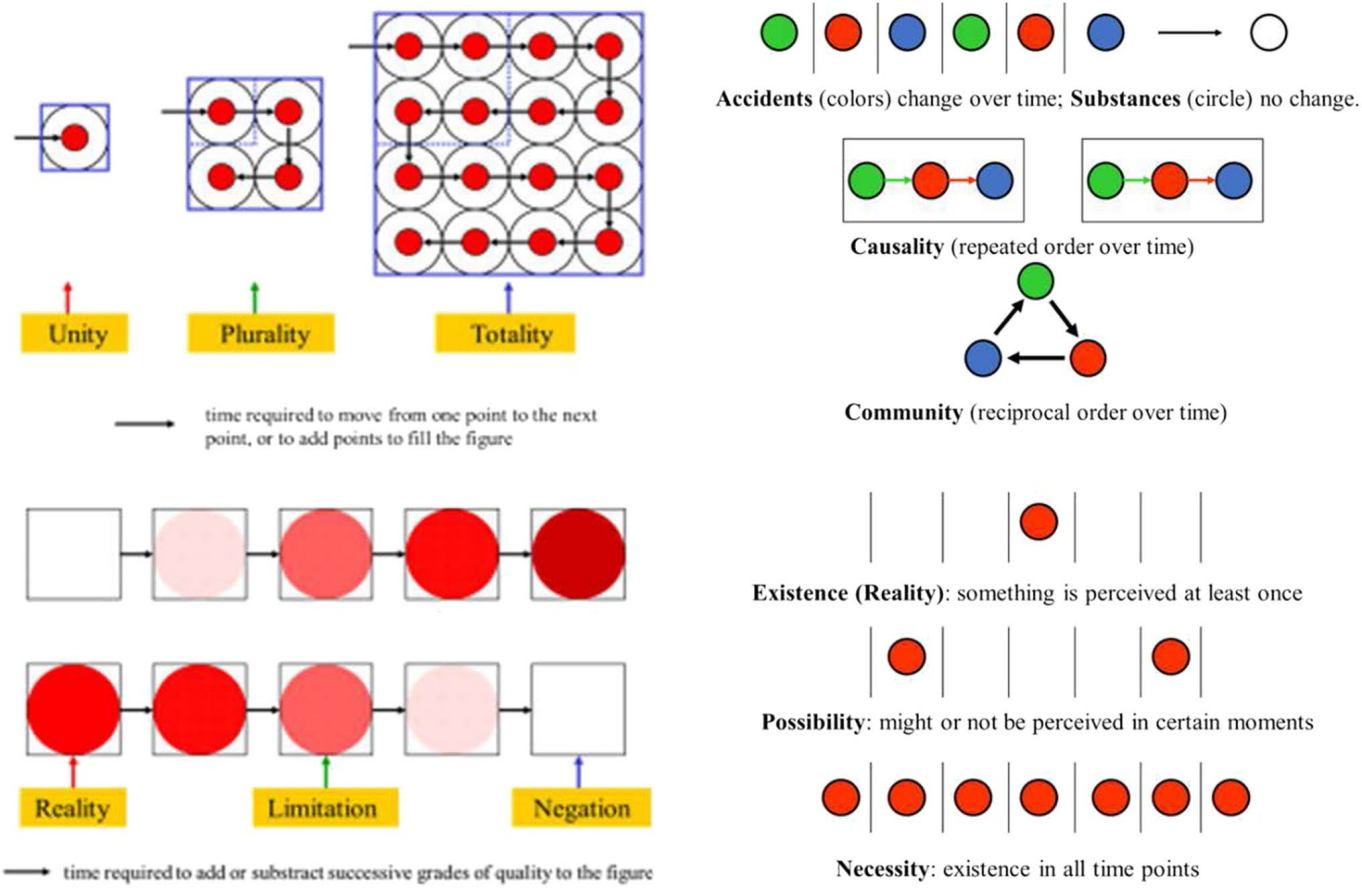
Extensive and intensive magnitudes, substances and necessities, revealed by time. The schemas are able to analyze the objects perceived (intuited) by the sensibility by using a “time dynamics” procedure. Left at the top, the time in filling a virtual space provides information about quantity (magnitude); at the bottom, along time, different qualities (intensities) are tested (color, in the figure) until reaching the one fitting with the empirical concept (reality) also informing about increase, limitation or absence of this quality. Right at the top, differentiation of accidental detection (colors changing over time) from detection of substances (the circle never change); at the bottom, detection of existence in time (occurs at least once), possibility (might or not occur at a given time), and necessity (necessarily occurs at all times)

For instance, the *Intensity* schema corresponds to the expression of *qualitative* categories when operating in the analysis of intuited objects. We can imagine that the “intensity” of the intuited object is evaluated by something like a “scanning” process, measuring the *time* required to fit with the “intensity of the quality in the object” when the category successively compares the intuited object with a series of empirical concepts ordered in a succession of different intensities (Fig. 5a). “No intensity” could correspond to the Negation category; “full-intensity” to the Reality category (affirmation); and “intermediate intensity” to the Limitation category. Similarly, the *Magnitude* schema corresponds to the expression of *quantitative* categories when analyzing a given type of intuition. The “magnitude” of the intuited objects could also derive from a time analysis, using a time series of empirical concepts, for instance in the form of dimensional points. In a sense, the “magnitude” can be measured by the time required to consider the object through the virtual addition (or subtraction) of time points. If a single “period of time” is used to add a single point, it corresponds to the Unity category; if several periods are used, they correspond to the Plurality category, and when no more periods of time could be added to cover the object, we refer to the Totality category (Fig. 5b).

Each of the relational categories has its own schema. The Inherence category has the *Permanence in time* schema: substances are permanent over time, accidents change over time. The *Repeated order in time* schema corresponds to the Causality category: if a particular sequence is constantly followed in actual experience, the first component of the series probably determines the second (cause-effect). The *Reciprocal action* schema corresponds to the Community category if, in a given time point, only a single type of intuited object (and never the opposite) occurs, and vice versa.

Similarly, a particular schema corresponds to each modal category. the *Existence at least in a time point* schema corresponds to the Reality category: the intuition has been experienced at least in one time (and the contrary intuition was then excluded at the same time). The *Existence in some time points and not in others* schema corresponds to the Possibility category. Lastly, the *Existence in all times* schema corresponds to the Necessity category.

## Quality-control of the analytic process: the principles of cognition

The principles of cognition are discussed in CPR in the obscure chapter entitled “System of all principles of the pure understanding” (A147/B187). In this chapter, Kant envisages the possibility of establishing the general “a priori” conditions under which the faculty of judgement is *justified* in using the pure concepts of understanding (the categories) to produce judgements. In the global architectural frame of the Theory of Knowledge, we might locate the principles of cognition as a checkpoint, a quality control of the entire process of the Analytic compartment. Kant’s designations for these principles of cognition are intimidating and confusing and do not clearly explain what those principles really mean. There are four principles corresponding to the four groups of categories. The *Axioms of Intuition* correspond to the “qualitative” categories, which state that any intuition as object of understanding should have an extensive magnitude, should be wrapped in space and time. The *Anticipations of Perception* correspond to the “quantitative” categories, indicating that all intuitions should have a degree, that is, a given intensity (if the intensity is zero, the intuitions do not exist). The *Analogies of experience* correspond to the “relational” categories, meaning that what is intuited should be inserted in a simultaneous relational frame; that is, the experience is possible only if a link can be established between perceptions, as anything perceived by experience is necessarily related. Lastly, the *Postulates of empirical thought in general* correspond to the “modal” categories and state that the intuition should conform to the conditions of the experience (the previous principles), should be real (perceived by experience), and eventually, necessary (existing accordingly to a law).

## The third compartment: dialectics

**Fig. 6:**
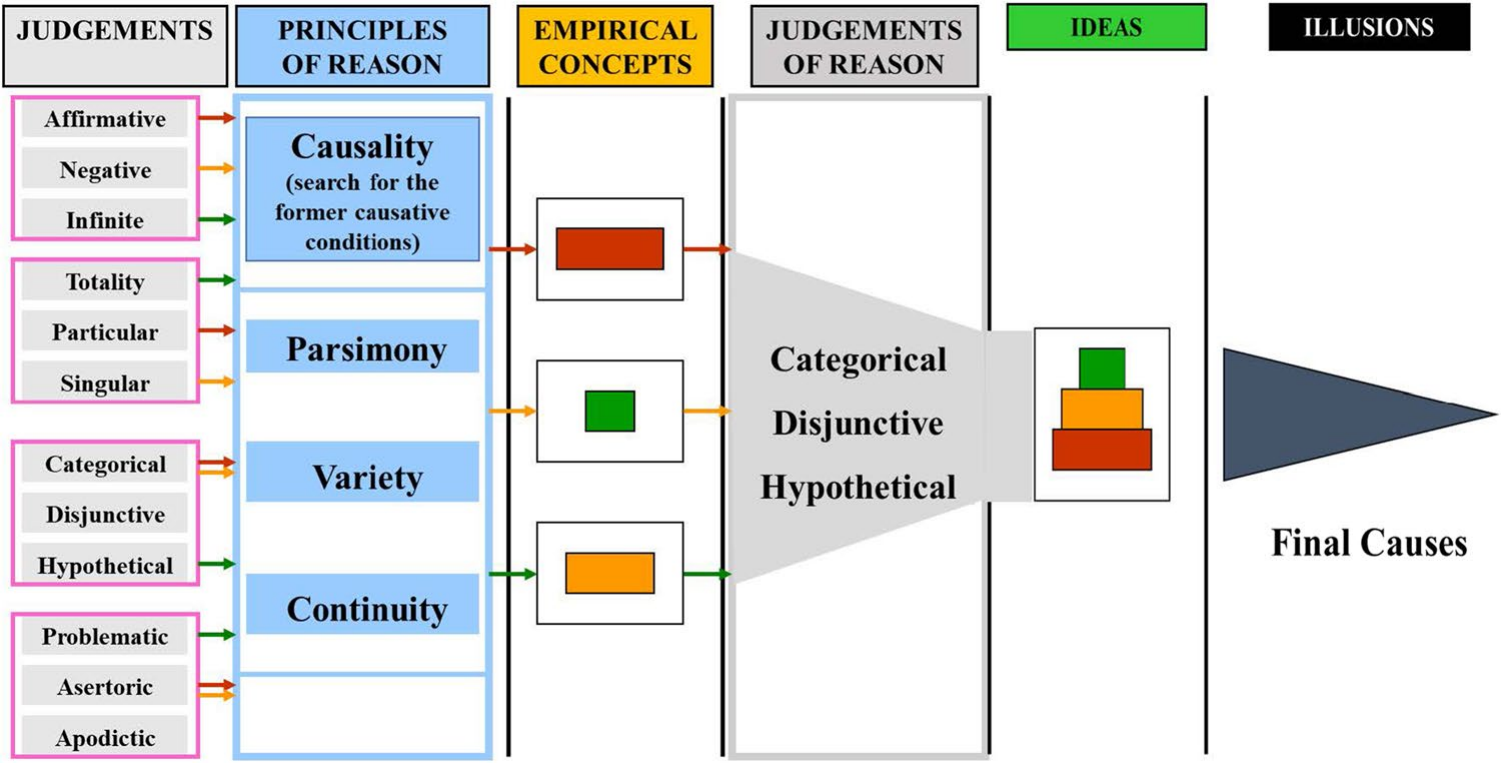
The process of understanding (knowledge). Once the objects of intuition captured by our sensibility have given rise to analytic judgements (grey), the process of reasoning occurs by a progressive condensation of knowledge using the principles of reason (blue), able to identify the empirical concepts, that are combined by judgements of reason (dark grey) to give rise to synthetic knowledge (ideas, in green). The progressive condensation of the elements of knowledge seems to be “attracted” by final (or pseudo-final) causes (illusions), that are necessarily out of the knowledge process. The Kant’s process of knowing, with successive refining and assembling steps recalls an evolutionary process searching for a final optimum of complex information

Dialectics is the compartment of reason, that is, the faculty of linking judgements in a synthetic process following a mysterious “a priori,” transcendental attraction to reduce and condense in a small number of principles the multiplicity of knowledge generated during the process of understanding (Fig. 6).

This attraction for rational synthesis is based on the **causal Principles of Reason**, which state that everything has a cause, that its existence is determined by some other thing. In the limit, the reason seeks for the ultimate, unconditioned cause (B364); its dialectic, its movement, aims to investigate the absolute, unconditioned knowledge. The Principles constitute a highly abstract concept. We can imagine the Principles as a type of virtual screen or imaginary focus beyond a spherical mirror, nothing in itself but able to connect to a kind of representation provided by the judgement process. The condensing function of these unconditioned **Principles of Reason** originate new concepts, independent from those emerging from aesthetics and analytics (A299/B355). These new concepts are now “thinkable” (objects for reasoning) and correspond to “transcendental ideas” (A311/B368) or “ideas of reason” (A669/B697). Three Principles or maxims are applied to ensure the correct synthetic activity. The principle of **parsimony** states Occam’s razor “*non sunt multiplicanda entia sine necessitate*” (entities are not to be multiplied without necessity). The principle of **variety** states that the diversity of beings should be preserved. The principle of **continuity** states that the logical cause-effect continuum between beings cannot be violated, remembering the basic Leibnizian concept that “*natura non facit saltus*” (nature does not make jumps).

Everything in Dialectics is based on the progressive linkages on the pure concepts of reason, leading to **Judgements of Reason**. Judgements acting on/producing relationships between judgements are in fact a recapitulation of the function of “relational” categories. As previously stated, these are **categorical** judgements (A is the *substance* of B, there is no difference), **hypothetical** judgements (answering the question “Can we attribute to a given temporal succession of intuitions A and B a cause-effect of the type ‘A produces B?’”) and **disjunctive** judgements A is either A or B (could be called a *divergency* judgement).

Forced by this knowledge dynamic seeking for the final cause (the one that explains everything), reason is compelled to follow what biologists could define as a “phylogenetic” process of synthetic understanding, always requesting these primary causes. At each step of knowledge, judgements are used as premises in a syllogism, and conclusions are obtained by reason. These conclusions are then incorporated as premises in new syllogisms, determining a *ratiocinatio polysyllogistica* (A331) of indeterminate length (B387). “General knowledge may serve as the major (premise) in an inference” (A300/B356) and is therefore converted into a new principle, that is, knowledge that is used to build new knowledge. This new knowledge is, in a sense, “internal novelties,” “internal objects” that are presented to reason, with a closer or remoter causal root in empiric knowledge but born in reason itself and therefore non-empirical. Interestingly, Kant finds a resemblance of these “internal novelties” generated by inference in the process of understanding and the intuitions, perceived “external novelties” that were presented to our sensibility. At the same time, as they serve to link premises forming new knowledge, they also might resemble categories. If categories are “pure concepts of understanding,” these “pure concepts of reason” are **Transcendental Ideas or Ideas of Pure Reason** (A311-A312; A669-B697). There Transcendental Ideas require the unity of the thinking subject, the unity of thinking conditions, and the absolute unity provoked by the attraction of the final and highest concept of the “being of all beings” (A335-B392). The name “idea” is recovered from Plato, and Kant uses it to reinterpret the meaning of platonic ideas. In Kant’s view, the platonic ideas were archetypes of the things themselves; whereas in Kantian doctrine, because of his “Copernican revolution,” the ideas, as a late consequence of an imprint of the reality in our sensibility, are just devoted from any direct link with the “external objects.” The function of transcendental ideas is “regulative,” that is, they serve to link judgements in an approachable manner without disturbing the higher possible synthesis of all particular knowledge offered in our process of understanding.

However, it is impossible to indefinitely pursue the series of causes pushing synthetic knowledge. Therefore, at which point will the full condensation of knowledge take place? The apparent last steps of the causal chain should necessarily be synthesized with the previous causes in a possible next step of understanding. These **provisional end-points** (as if they were the “final cause”) in the process of reasoning are also “principles of reason,” but clearly the whole synthetic process should be attracted by something that is beyond any cause, that is, unconditioned, the final cause.

Of particular interest for scientists is that emergence from hypothetical judgements (cause-effect linkages) of **transcendental ideas, nature** (the world), **the unconditioned limit of all series of causal events**, and the “absolute series” expressing the unity of the series of conditions leading to empirical evidence. Nature constitutes a transcendental idea attracting knowledge of everything that is caused, but “the nature of nature” remains undetermined, or better stated, cannot be determined, given that nature does not correspond to anything, even if we use this term “as if it corresponded to something,” a **transcendental illusion**. Note that Kant’s main message in the Critique is that human reason should only deal with “experiences” and that any inclination of reason beyond the limits provided by empirical objects constitute a source of **illusory for knowledge**, which can only be operatively used as a virtual (operative) attractor. We can recognize in ourselves a kind of illusory shadow of these last causes (as “nature”), our irrepressible curiosity of knowing, a curiosity, as an avatar of the “last cause” provoking knowledge, that can be modulated by education.

The main focus of the present perspective is aimed at disseminating among biologists, and particularly among microbiologists (“the basic biologists”), Kant’s Theory of Knowledge as presented in the *Critique of Pure Reason*. A concise view of the meaning of this Kantian approach for microbiologists is presented in Box 1. However, Kant developed his main concepts about biological phenomena in the two Introductions and the second half of the *Critique of the Power of Judgment*, in which he discusses the peculiar and complex organization of living nature, “not analogous with any causality that we know,” given that there is a mysterious “attraction” where the final cause influences, in his point of view, the connections among efficient causes. To analyze this part of Kantian philosophy is out of the scope of this publication, and we would like only to remark on, in the next paragraph, the conceptual link of the Theory of Knowledge with Evolutionary Theory.

Box 1 Knowledge in Microbiology: a concise Kantian view

**Table:** 

Aesthetics
• What are the objects detected by our sensitivity (Aesthetics). That depends on our analytic technology. Before optical microscopy (Leeuwenhoek 1674) microbes were outside our sensitivity; before X-ray crystallography the DNA double helix remained in the dark; (Watson and Crick 1953); before electronic microscopy ribosomes (George E. Palade 1955) were outside our knowledge. These analytic discoveries make it possible to detect discrete “objects” of nature and assess them with our reason
**Analytics**
• The “objects” provided by Aesthetics should be “conceptualized.” There are microorganisms, but how different they are they from each other? By using Kantian categories, hierarchical classification allows us to link what is similar and to separate that which is dissimilar, as well as to provide “connections” in a qualitative, quantitative, relational, and modal (possibility, actuality) way. Fusing analytics with aesthetics, we reach “schemas,” where we understand the size, construction, and structure (such as the genetic code, the genome sequence), permanence in time, or compatibility in terms of the various microbes or their reciprocal interactions. Such knowledge should be real (accessed by experience) or even necessary (required by the logic of the real world)
**Dialectics**
• Aesthetics and Analytics have informed our reasoning about microbial organisms, but dialectics forces us to think (ideas of reason) about the causal processes that might explain their existence “as they are.” The Principles of Reason induce thinking about how diversity has evolved and how compatible variation is (“variety states”) with continuity and parsimony. Dialectics might re-propose new objects to the aesthetics: “are there objects nested inside other objects?” which is implicit in the ideas of the Units of Selection and the Evolutionary Transitions (Samir Okasha and John Maynard-Smith 1995). Is there, as we can imagine in evolutionary biology, a chain of causes driven by a transcendental attraction in nature for a kind of entangled unity, encompassing not only biological entities, but the whole world? (Lovelock and Lynn Margulis, mid 1970’s)

### The Kant’s theory of scientific knowledge and the evolutionary theory

Did Kant’s Theory of Knowledge influence the scientific climate that gave birth to the Theory of Evolution? As commented by Ernst Mayr, “Considering the seemingly universality of evolutionary thinking in Germany during the first half of the nineteenth century, it is quite puzzling that this background did not lead to the elaboration of a substantial theory of evolution by even a single German biologist” (Mayr 1982). Why was there no German Darwin? The local powerful Linnaean influence of essentialism (there is no way by which an essence, a single substance or species cannot be converted into another) was shadowing the clearly evolutionary dynamics of the consecution of knowledge in Kant’s Theory of Scientific Knowledge, where judgements act as selective events orientating the progress of elementary pieces of knowledge towards the (always partial) truth. A number of unselected empirical entities of nature enter into the compartment of sensibility and, once converted into elementary intuitions, are submitted to the combinatory effect of the analytic compartment and subjected to a progressive system of judgement barriers that is allowed to persist only if a number of principles are fulfilled. The surviving elements are those whose properties ensure the elements possible integration with other elements in synthetic judgements progressing to the truth. The truth is the highest fitness in the landscape of possible knowledge, not implying accordingly to Kant any “evidence” (implicit in Descartes’ though) of a complete correspondence between understanding (theory) and reality (3). Certainly Kant might have been a forerunner of Darwin if the gradualist biological causal-effect bases had been available in Kant’s time. Indeed, Kant’s “A General History of Nature and a Theory of the Heavens” (1755) includes gradualistic views: “The future succession of time, by which eternity is unexhausted, will entirely animate the whole range of space…. and will *gradually* put it into a regular order with is conformable with His plan… the creation is never finished or complete. It did once have a beginning, but will never cease.” The concept of a creative function of time (“the future succession of time…will entirely animate…”) pushing evolution is an interesting, albeit untestable hypothesis (Baquero 2005). For Kant, all thinking processes are also biological processes, and there is a (non-explicit) correspondence between knowing and evolving (Hanna 2006) which does not imply any teleological trend, except if teleology is understood in heuristically (a tool facilitating an approximation to a possible reality). As in Kant`s process of knowing, evolution can be considered as an anti-entropic process leading a progressive condensation of information, increasing fitness as information is energy (Toyabe et al. 2010).

In fact, this approach is currently considered in the contemporary philosophy of biology and theoretical biology (Gambarotto and Nahas 2022). If Kant’s Theory of Knowledge resembles the natural evolutionary process, it implies that our mind, our “knowing machine” acts similarly (and might be influenced) by evolution. This important concept is probably the cornerstone of modern evolutionary epistemology and is closely related to science education.

### Education in microbiological sciences and evolutionary epistemology

Working in the lab or with computer bioinformatic programs, undergoing training in novel technologies, and reading publications in one’s field of interest are certainly necessary activities for scientists but are not sufficient. The essential element is thinking, being involved in free, personal thinking. Science education should therefore include education far beyond technology. Increasing the faculty of understanding is a key educational target, although it does not, in and of itself, help derive explanations for phenomena as does not in itself help to acquire explanations of the phenomena, but can project such understanding in a practically usable form (Lorenz 2009). Education for thinking in science can (but not necessarily) be oriented towards developing particular “objectives of knowledge,” as has been proposed (Haviz and Maris 2020; Molefe and Aubin 2021).

Evolutionary epistemology is a term coined by Campbell (1974), using the analogy of knowing and evolving, and has deeply influenced education in Science (Gontier and Bradie 2021; Bradie and Harms 2020). Knowledge deploys experimental facts, models, metaphors, and theories that (as living beings) are subjected to the continuous judgement (critique, selection) of science, and only the fitter conceptual changes tend to survive and diversify, serving as new growing points enriching the connections between varied fields of knowledge, “patches of knowledge.” Indeed, ecosystem-based thinking in science mimics an evolutionary process, so that genetic or organismal coalitions and interactions give rise to emergent evolutionary properties, i.e., “unexpected novel knowledge” (Bapteste et al. 2012).

The “knowledge machinery” proposed by Kant in his CPR should also be the result of evolution, and the innate capacities of our understanding, given that the “a priori” Kantian concepts were probably born in non-human organisms (Lorenz 2009; Ruse 2009). This is an “Evolutionary Biology of Reason” where Kant’s knowledge construction laws emerge as an intrinsic aspect of evolutionary biology (Cooper 2003), a field certainly close to evolutionary epistemology. Compared with the rate of scientific progress (knowledge), the progress of evolutionary biology of the knowledge machinery provided by reason is probably negligible (Ruse 2009), which is likely due to the progress in education, availability of information, and, in general, in human cultural networking. An open question for philosophical and scientific research is the future role of computer sciences, including artificial intelligence and machine learning, as an epistemological complement to advance the possibilities for developing human knowledge and understanding. Science, is the knowledge of most worth (Liu et al. 2017). and should be understood as such by students, even undergraduates (Hoskins and Gottesman 2018).

Our intention in the precedent paragraphs was more to capture in a number of images (the figures) the spirit of Kant’s Theory of Scientific Knowledge rather than describe in detail the complexity of Kantian thoughts. The extent to which the author might succeed in such a goal must be measured by the degree of stimulation of at least some students of natural sciences in reconstructing bridges between philosophy and experimental biological sciences. Indeed, that also imply a reflection on the conceptual roots of Microbiology as a Science.

### The roots of microbiology as science and Immanuel Kant: from taxonomy to synthetic biology

Beyond the influence of evolutionary thinking, there are “classic” and “modern” fundamental aspects of microbiology where Kant’s shadow can be recognized.

Among the “classics,” an important task of microbiology is the recognition of microbiological entities. Bacterial systematics is involved in the establishment of the difficult-to-grasp objects of taxonomy, particularly the species taxon. Around 1850, this problem had not yet been addressed, and Ferdinand Cohn (1828–1898), a mentor of Robert Koch and corresponding with Charles Darwin, considered that in the field of bacteriological systematics “One has to start at point zero” (Drews 2000). In the revision of the International Code of Nomenclature of Bacteria in 1975, considerations were made regarding whether a classification of bacteria does justice to the laws of homogeneity, specification, and continuity as laid down by Kant in his transcendental dialectic in the *Critique of Pure Reason* (CPR). Phylogeny as a way of classifying (judging) entities accordingly with pure reason was certainly considered by Kant (Lerussi 2012). The variety of infra-subspecific subdivisions was taken into consideration, but the species maintained a preferential position. Kant proposed that biological entities tend to preserve their internal unintentional purposive organization at the level of species, but they can be modified by external changes (26). The species taxon was also maintained because of the needs of communication in applied bacteriology, also in agreement with Kant’s postulates (Beckman and Jochensen 2022, Habs 1981). Note that Kant’s theory of knowledge has a final moral purpose, which is to avoid mistakes in order to ensure “the use and benefit of man’s life,” which suggests an applied, practical, and humanitarian objective.

Among the “modern” aspects of microbiology where we can find Kant’s roots is in the synthetic biology of microorganisms. How mechanical phenomena can result in biological phenomena remains a fundamental Kantian question regarding the origin and evolution of life (29). Certainly, living beings have a “mechanical background” but it is extremely complex, subject to variation, and therefore impossible to describe, at least at the level of what can be known in physics (Baquero 1977). The essence of a living entity cannot be understood by merely studying its parts (Kim et al. 2021). Moya et al. (2009) appropriately quoted the famous Kantian expression: “There will never be a Newton of a grass blade,” given that in living entities, every part is a function of the whole and the whole is a function of every part, and in which “nothing is for nothing.” Therefore, the Kantian prediction is that knowledge based only on the component parts will be worthless to understand life (Moya et al. 2009). In microbiology, the only way out is synthetic biology, which is an “epistemological methodology” based on the combination of parts of biological systems to gain partial but cumulative judgement-based knowledge about cellular organization and the collective behavior of the microbiosphere, without intending to reach a final, transcendental explanation. Note how close this approach is to the “technology (legislation) for knowing” proposed in Kant’s CPR.

Finally, Kant’s philosophical heritage contributed to the recognition of microbiology (with biological and evolutionary natural sciences) as specific sciences. Recapitulating the Ernst Mayr idea that biology as a science, that is, developed from empiric (scientific) knowledge, is based on concepts rather than laws. Knowledge of biological entities should also be based more on empirical concepts, not on laws established by philosophers, even if Kant proposed that “the philosopher is the legislator of human reason” (A839/B867). These laws are of *reason*, not necessarily of nature. Biological sciences, such as microbiology, differ from other sciences because of nature`s apparently logical but unintentional purposiveness, which produces in the philosopher a feeling of admiration (Stoner 2019) given that this characteristic does not comply with the closed legislation of Kantian pure reason. However, our knowledge of biology should necessarily follow reason, but understanding that biological entities always point beyond themselves, a “cause of admiration.” This is the transcendental admiration and even reverence that a wise man such as as Josep Casadesús experienced when benching, teaching, and thinking about microbes.

## Data Availability

No applicable.
